# Supplementary material for: A New Lineage of Perch Rhabdovirus Associated with Mortalities of Farmed Perch
Source: Pathogens. 2021 Sep 28;10(10):1256. doi: 10.3390/pathogens10101256 (PMC8540231; doi:10.3390/pathogens10101256)
Supplement: Supplementary file 1 [file pathogens-10-01256-s001.zip › pathogens-1365951-supplementary.pdf]

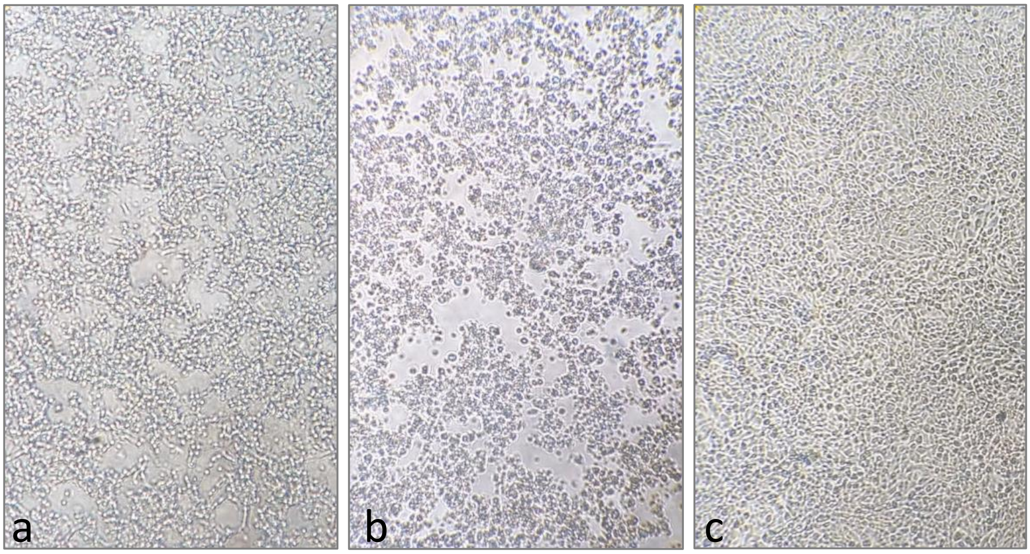

Suppplementary Figure S1. Cytopathic effect associated with the perhabdovirus 20-43. Cells are BF2 observed several days after infection (pi) (**A**) 4.5 days pi. (**B**) 5.5 days pi. (**C)** negative control 4.5 days pi.

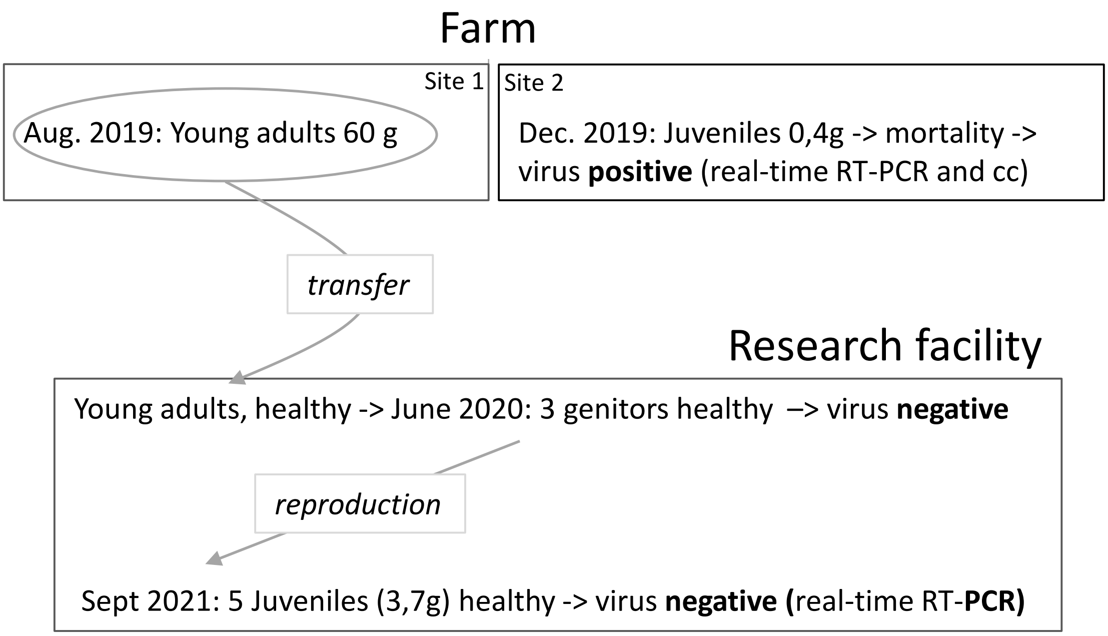

Supplementary Figure S2. Sampling of the fish.

Name	Target	Sequence (5'->3')
oPVP116	G	ACWTGTGAYTWCMGWTGGTATGG
oPVP118	G	CTGTTAGCTGTTTTTTTCATA
oPVP126	G	GATATGAAAAAAACTGCAACAG
oPVP141	N	GAWWTCTGTTAAAGTTTTTTTC
oPVP143	N	TGGATATGTTCTACTTCCACTTCA
oPVP529	N	GTGCAGGAARTCACCATACTCATC
oPVP530	N	CAGCAGAGCACAGGTCATTTG
oPVP705	5' end	GCAACCATTGGAAGTGTGAT
oPVP706	5' end	ACGAGAAAAACCACACCAGA
oPVP707	5' end	CGACCATTGGGAGTGTGAT
oPVP714	N	ACGAGAAAAACCAMACCAAG
oPVP715	N	GGTGGCAACTCTCACTACTG
oPVP716	G	GTGCAGGCAATTCTCC
oPVP717	N	ATCAYCATRGCRAYAGGGTC
tqPVP30	N	FAM-TCTGCCAATCCTGGAGTTCACTGCTG -BHQ1

Supplementary Table S1. Primers and probe used in this study.
